# The Effects of Group and Home-Based Exercise Programs in Elderly with Sarcopenia: A Randomized Controlled Trial

**DOI:** 10.3390/jcm7120480

**Published:** 2018-11-26

**Authors:** Maria Tsekoura, Evdokia Billis, Elias Tsepis, Zacharias Dimitriadis, Charalampos Matzaroglou, Minos Tyllianakis, Elias Panagiotopoulos, John Gliatis

**Affiliations:** 1 Department of Physiotherapy, School of Health and Welfare, Technological Educational Institute (TEI) of Western Greece, 25100 Aigio, Greece; ebillis@teiwest.gr (E.B.); tsepis@teiwest.gr (E.T.); orthopatras@yahoo.gr (C.M.); 2 Department of Orthopaedics, School of Medicine, University of Patras, 265 04 Patra, Greece; mintylli@med.upatras.gr (M.T.); ecpanagi@med.upatras.gr (E.P.); gliatis@hotmail.com (J.G.); 3 Department of Physiotherapy, General University Hospital Attikon, 12462 Athens, Greece; zachariasd@hotmail.com; 4 Rehabilitation Clinic, Department of Medicine, University of Patras, 265 04 Patra, Greece

**Keywords:** sarcopenia, exercise, group exercise, home exercise

## Abstract

Physical exercise is effective for sarcopenic elderly but evidence for the most effective mode of exercise is conflicting. The objective of this study was to investigate the effects of a three-month group-based versus home-based exercise program on muscular, functional/physical performance and quality of life (QoL) across elderly with sarcopenia. 54 elderly (47 women, 7 men aged 72.87 ± 7 years) were randomly assigned to one of three interventions: supervised group (*n* = 18), individualized home-based exercise (*n* = 18) and control group (*n* = 18). Body composition was determined by bioelectrical impedance analysis, calf measurement with inelastic tape and strength assessments (grip and knee muscle strength) via hand-held and isokinetic dynamometers. Functional assessments included four-meter (4 m), Τimed-Up and Go (TUG) and chair stand (CS) tests. QoL was assessed with Greek Sarcopenia Quality of Life (SarQol_GR) questionnaire. Outcomes were assessed at baseline, immediately post-intervention (week 12), and 3 months post-intervention (week 24). Significant group x time interactions (*p* < 0.001) were observed in QoL, calf circumference, TUG, CS, and 4 m tests, grip and knee muscle strength. Group-based compared to home-based exercise yielded significant improvements (*p* < 0.05) in muscle mass index, CS and 4 m tests, calf circumference, muscle strength at 12 weeks. Most improvements at 24 weeks were reported with grouped exercise. No changes were found across the control group. Results suggest group-based exercise was more effective than home-based for improving functional performance.

## 1. Introduction

Sarcopenia, defined as low muscle mass and low muscle function and/or reduced physical performance [1] is a major public health consideration [2,3]. It leads to serious adverse health consequences such as functional disabilities, frailty, fatigue, falls, fractures, hospitalizations, multiple comorbidities (osteoporosis, diabetes mellitus) [4], mortality, and compromised quality of life [3,5,6]. Exercise programs and nutritional approaches are considered to be the main interventions for managing sarcopenia [7]. Although exercise seems to have the most beneficial effects [8,9,10,11,12,13,14,15,16], exercise programs are highly variable, in terms of type (resistance, aerobic, multicomponent, etc.) and training mode (type, frequency duration, setting, etc.). Furthermore, limited evidence exists regarding exercise intervention as monotherapy [12,13,14,15]; thus, limiting the effects of exercise alone. Four systematic reviews [1,17,18,19] published in recent years on different nonpharmacological treatments for sarcopenia highlighted the diversity across studies in terms of the samples’ nutritional status, degree of physical frailty, the exercise training mode as well as the outcome measures used. Additionally, quality of evidence was rather low [18]. And, although exercise has positive effects in improving muscle strength and physical function, especially when 3 months’ resistance exercise programs are administered [7], the current subject merits further research.

Regarding the mode of exercise, an important factor is whether it is performed individually or in a group. Although both exercise deliveries are cost-effective [20], supervised group-based exercise programs appear superior in older participants for facilitating strong adaptations to exercise (muscles, bones, and cardiovascular system). Yet, they are associated with difficulties regarding patient adherence (accessibility issues of center-based programs). Alternatively, literature supports home-based exercise programs, in that, once patients are committed to them, they have long-term benefits [20,21]. Effects of center-based versus home-based exercise programs indicate variability in their results across different patient groups and populations [22]; suggesting a need to further explore the effectiveness of these delivery options. No study so far has evaluated group versus home-based exercise programs for elderly with sarcopenia [23].

The purpose of this study was to compare the efficacy of a group-based exercise training program over a home-based exercise training program on muscle strength, muscle mass, physical performance, functional status and quality of life parameters in elderly people with sarcopenia living in Greece.

## 2. Experimental Section

### 2.1. Participants/Study Population

Participants were recruited from the region of Achaia, mainland in Western Greece using flyers, posters, and advertisements in newsletters. The assessment procedure was carried out at three sites; University Hospital of Patras, Technological Educational Institute (TEI) of Western Greece, and 2nd Open Care Centre of Patras for the Elderly.

Before recruitment, each participant was screened for eligibility whether he/she met the criteria for sarcopenia. Eligible were patients who were defined as having presarcopenia, sarcopenia, or severe sarcopenia. According to the criteria reported by the European Working Group of Sarcopenia in Older People (EWGSOP), presarcopenia is characterized by low muscle mass, sarcopenia is characterized by low muscle mass, plus low muscle strength, or low physical performance, and severe sarcopenia by low muscle mass, low muscle strength, and low physical performance [24]. All patients defined in any of these categories could participate in the study. Additional criteria for inclusion were age 60 years or older and living independently in the community. Exclusion criteria included (a) cognitive impairments, (b) neurological disorders, (c) pacemaker fitted, (d) cardiovascular diseases or high blood pressure not controlled with medication, (e) previous surgery on lower limbs affecting gait, (f) medical or other musculoskeletal problems that could affect ability to complete objective assessments, (g) currently engaged in exercise training (within last 3 months), and (h) body mass index (ΒΜΙ) > 50.

All participants signed an informed consent form prior to their inclusion. The protocol was approved by the Ethical Committee of the TEI of Western Greece (reference number 4052). Additionally, the study has been registered at www.isrctn.com following identification number: ISRCTN92538100.

### 2.2. Randomized Group Assignment

After the baseline assessment, subjects were randomly assigned to one of three intervention groups: (i) supervised group exercise, (ii) individualized home-based exercise group, and (iii) control group. Randomization was undertaken by the use of consecutively marked and sealed envelopes. A researcher generated the random allocation sequence in blocks of 6 and prepared the sealed envelopes for the total population [25]. Numbered envelopes for the total population were handled by an independent researcher who did not participate in any of the interventions.

### 2.3. Outcome Measures

All participants were assessed at baseline, immediately post-intervention (week 12), and at 3 months post-intervention (week 24). Measures included an interview survey, body composition assessments, muscle strength assessments, physical function tests, and quality of life assessment. All measurements were made by the same health professional, a geriatric physiotherapist, highly experienced in using the aforementioned measurements.

#### 2.3.1. Interview Survey

Each participant was interviewed face-to-face to assess his/her history of falls, lifestyle habits, exercise habits, and medication use. Participants also completed a Mental State Examination (MMSE), which consisted of a 30-point questionnaire for evaluating their cognitive function. MMSE has been validated and extensively used in both clinical practice and research [26,27].

#### 2.3.2. Body Composition Assessment

Height was measured with a wall stadiometer without shoes. Body weight was measured to the nearest 0.1 kg and height was measured to the nearest 0.1 cm. Measurements of height and weight were used to calculate body mass index (BMI) (kg/m^2^). Body composition was determined using bioelectrical impedance analysis (BIA), with a Tanita BC-601 model body analysis monitor. Participants removed their socks, stood on two metallic electrodes on the floor scale barefoot, and held two metallic grip electrodes placed in the palm of their hand with their fingers wrapped around the handrails. Fat free mass (FFM) was measured by BIA and skeletal muscle mass (SMM) was calculated by the following equation: SMM (kg) = 0.566 × FFM. Skeletal muscle mass index (SMMI) was calculated as skeletal muscle mass (kg)/height squared [26,28]. Cut-off thresholds for skeletal muscle mass indices were set at 7.23 kg/m^2^ and 5.67 kg/m^2^ in males and females, respectively [24]. Participants were recommended to have a bowel movement within 30 min before the measurement, and also not consume any alcoholic beverages and meals for at least 48 h and 4 h, respectively before the tests [29].

Calf circumference (CC) was measured with inelastic tape with the elderly participants in the upright position, with feet 20 cm apart. CC was measured at the calf’s greatest girth [30,31,32].

#### 2.3.3. Muscular Strength Assessments

Handgrip strength (HGS) was measured using a standard hydraulic hand dynamometer (Saehan, Seoul, Korea) and according to EWGSOP, the cut-off thresholds for handgrip strength are 20 kilograms (kg) for women and 30 kg for men. Grip strength measurement is a valid and reliable method for measuring muscle strength [33]. Each patient’s dominant hand was tested. The patients were seated with the arms adducted, the forearm to be tested unsupported, elbow flexed at 90°, and wrist in a neutral position. Participants were asked to apply the maximum grip strength for 3 times and the highest value was recorded as the subject’s grip strength.

Knee Muscle strength was assessed with Biodex isokinetic dynamometer for knee flexors and extensors (isokinetic concentric extension-flexion at 90°/s and 180°/s). All measurements were carried out by the same (trained in this procedure) physiotherapist. Participants were asked to perform the movement with their maximal strength, while verbal encouragement was offered throughout the effort. Calibration of the equipment was performed according to the manufacturer’s specifications before every testing session.

#### 2.3.4. Functional Assessments

Walking speed was assessed via the four meter (4 m) walking test. They were informed to walk 4 m with their usual speed. Function was assessed using the Timed Up and Go test (TUG) and the chair stand (CS) test. For both functional tests (TUG and CS test), participants were seated in a standard 45 cm height chair, with the back against the chair, both arms resting along their body and both feet completely resting on the floor. For the TUG assessment they were instructed, on the word “go,” to get up and walk 3 m forward, as fast as possible, turn around an obstacle, return to the chair, and sit down again [34]. For the CS test participants needed to stand up 5 times from sitting (while keeping the arms crossed) and sit down again as fast as possible [35]. Procedures were fully explained before assessment followed by a familiarization attempt. For all three functional tests, time in seconds was the recorded variable.

#### 2.3.5. Quality of Life Assessment

Quality of life (QoL) was assessed using the Greek validated version of the Sarcopenia Quality of Life (SarQol_GR) questionnaire [29]. This questionnaire is the first sarcopenic-specific, self-administrated QoL questionnaire including 22 questions, rated on a 4-point Likert scale of frequency (often, sometimes, rarely, never) and intensity (a lot, moderately, a bit, not at all). SarQoL is organized into seven QoL domains: Physical and Mental Health, Locomotion, Body Composition, Functionality, Activities of Daily Living (ADL), Leisure activities, and Fears. The total scoring of the SarQoL questionnaire ranges from 0 (worst imaginable health) to 100 (best imaginable health) [36].

### 2.4. Interventions

Both exercise programs were delivered by a well-trained and highly-experienced geriatric physiotherapist. Participants were randomized into three groups; group-based exercise, home-based exercise and control group.

Group-based program: Participants attended a 60-min comprehensive progressive group exercise program twice a week for three months. In addition, they had to walk for 100 min per week (minimum; ~30–35 min; 3 times/week). The exercise session included a 5–10 min warm-up (predominantly stretching) of the neck, shoulders, lower back, hips, knees and ankles, 20–30-min of strengthening exercises, 20 min of balance and gait training exercises, followed by a 5–10 min cool-down. Strengthening exercises were carried out in a progressive sequence from the seated position (which provided a secure and stable position) to the standing position. Strengthening focused on hip extensors and abductors, knee flexors and extensors, and ankle dorsi and plantar flexors. Strengthening of the upper limp focused on shoulder flexors, extensors, abductors, and elbow flexors and extensors. The balance exercises contained exercises, such as one-leg stands, tandem stands, and tandem walking. Participants were initially instructed to complete up to one set of 8 repetitions for each type of exercise, which gradually increased to 12 repetitions, and up to two sets for each exercise. The intensity of the exercise was based on the Borg Rating of Perceived Exertion (RPE). This is a psychophysical, category scale measuring physical activity intensity level with ratings ranging from 6 (no exertion at all) to 20 (maximal exertion) [37]. An explanation of this rating scale was provided to participants prior to the intervention to ensure proper understanding of exercise intensity [38]. Strengthening exercises progressed by applying additional weight or increasing the number of repetitions. Balance exercises progressed from standing to holding on to a stable structure to perform the exercise independently of support. All exercise sessions were carried out in the 2nd Open Care Centre of Patras. Attendance of each participant was documented throughout all sessions.

Home-based program. Participants in this group received home therapeutic exercises for 12 weeks. Additionally they were instructed to walk for 100 min per week (minimum; ~30–35 min; 3 times/week). The home-based exercise program included the same exercises with Group A, consisting of stretching, muscle strengthening, balance and gait training of moderate intensity. Each participant received a detailed booklet outlining the program, free weights for the upper limp and ankle cuff weights at the initial session. For each participant, the physiotherapist supervising the home program visited each participant’s home to instruct him/her on how to perform exercise safely and properly and revisited each subject three more times, to make progressive adjustments to the exercise protocol. Each of these four visits in the first 2 months took approximately 1 h each. The physiotherapist also made four telephone calls across the first 12 weeks, in order to ensure exercise compliance and to pick-up any problems. In addition, participants were asked to keep an exercise diary and record the days they completed the program. All subjects undertook the same intensity (RPE) and progression of exercises as had been prescribed for the group-based program. Participants in both exercise groups were instructed not to perform any additional physical activities in these 12 weeks.

Table 1 summarizes the intervention programs in more detail, as well as the exercise progressions for both groups.

Control group: Following the baseline assessment, the participants received an educational leaflet about sarcopenia with advice on diet, lifestyle, and activity. Participants allocated in this group received no exercises during the time of the study.

## 3. Statistical Analysis

G* power was used for sample size calculation. Based on this and the algorithms provided by SPSS (which were used for the statistical analysis), the detection of a large effect size (i.e., *f* = 0.4) for the group*time interaction of the 2 way mixed ANOVA (3 groups, 3 measurements) requires at least 15 participants per group (*a* = 0.05, power = 0.80). The addition of 3 elderly per group was decided after discussions with the other research members to control for any dropouts. Therefore, 18 patients per group were recruited in order to increase the statistical power as well as to prevent problems resulting from a potential patient dropout.

The descriptive characteristics were presented as mean and standard deviation. One-way AΝΟVA was used to verify baseline differences between the groups. Within and between group differences with the interaction of group*time, for each dependent variable, were examined by using the two-way mixed (ANOVA). The effectiveness of each type of intervention was additionally examined by using repeated measures ANOVAs. Post-hoc analysis was conducted with Bonferroni tests. A one-way ANOVA test was used in order to determine the difference between groups. The two-tailed significance level was set at *p* ≤ 0.05 for all tests. SPSS 20.0 (SPSS, Chicago, IL, USA) was used for the statistical analysis.

## 4. Results

**Figure A1:**
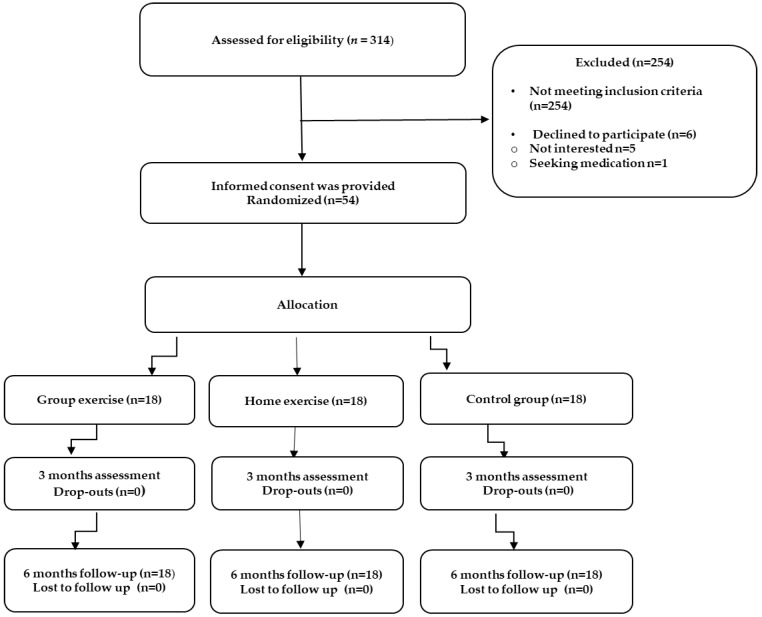
CONSORT flow diagram of patient selection and allocation.

Figure A1 presents the CONSORT flow diagram of patient selection and allocation in the present study. A total sample of 314 elderly people underwent baseline assessment and 60 (19.1%) participants were operationally defined as sarcopenic (Figure A1), based on the consensus criteria for the diagnosis of sarcopenia of the EWGSOP [1].

A total of fifty-four participants [men (*n* = 7) and women (*n* = 47)] with a mean age of 72.87 (SD = 7.02) years completed the study. No dropouts were recorded. Baseline parameters comprising the assessment for the clinical diagnosis of sarcopenia, as well as other population characteristics, were similar across groups (Table 2). The only significantly different characteristic across the groups was smoking. ΜMSE scores for all participants were between 28–30. Based on the results of the handgrip test, gait speed and the BIA measurements 45 (83%) subjects of the total study population were categorized as having sarcopenia and 9 (17%) as having severe sarcopenia.

There were no reported adverse events due to the exercise program, and those participants who completed the program had high adherence. The adherence rate for participants was 91.66% and 87.5% in the supervised and home-based exercise programs respectively, as calculated by dividing the number of exercise sessions of each participant, by the number of sessions they were expected to perform throughout the study.

Comparison between pre- and post- intervention changes in performance measures and body composition (at 12 and 24 weeks) (Table 3), showed significant group x time interactions in QoL (*p* < 0.001), CS test (*p* < 0.001), calf measurement (*p* < 0.001), HGS (*p* < 0.001), TUG (*p* < 0.001), SMMI (*p* = 0.003), right knee extension 90°/s (*p* = 0.003), right knee flexion 90°/s (*p* = 0.002), right knee extension 180°/s (*p* < 0.001), right knee flexion 180°/s (*p* < 0.001), left knee extension 90°/s (*p* = 0.03), left flexion 90°/s (*p* = 0.05) and left flexion 180°/s (*p* < 0.001). In terms of BMI there were no statistically significant differences across groups.

### 4.1. Within Group Differences

The group-based group had significant improvement in 15 out of 17 variables after 12 weeks of supervised group-based exercise training (all except for BMI and left knee strength in extension 180°/s). In terms of quality of life (SarQoL) subscores, statistical significant differences were recorded in 4 domains: ‘Locomotion’ (*p* = 0.006), ‘Functionality’ (*p* = 0.012), ‘Activities of Daily Living’ (*p* = 0.00), and ‘Fears’ (*p* = 0.013).

The home-based group improved significantly in 7 out of 17 variables at 12 weeks (TUG, Gait Speed, 4 m test, CS test, QOL, knee muscle strength-right knee flexion 180°/s, right knee extension 90°/s, and left knee flexion 180°/s). For SarQoL statistical significant differences were recorded in domains: ‘Functionality’ (*p* = 0.002) and ‘Activities of Daily Living’ (*p* = 0.00).

At 24 weeks, the group-based group yielded significant improvements in 11 out of 17 variables (SMMI, HGS, gait speed, TUG, 4 m test, and knee muscle strength), whereas, the home-based group improved significantly in only 3 out of 17 variables (TUG, 4 m test and gait speed), (Table 4). The Control group did not yield any significant changes in any variable at 12 or 24 weeks follow-up.

### 4.2. Group Differences

One way ANOVA analysis was conducted to examine the relationship between the three groups (Group-based, Home-based, and Control Group). Overall, both exercise groups improved in all variables compared to the control group. Consistently greater improvements in mean scores favored the group-based program compared to the home-based program (at 12 weeks) for SMMI, HGS, CS test, 4 m test, CC and knee muscle strength (right knee flexion 180°/s and left knee flexion 90°/s) (Table 5). In terms of quality of life, the SarQoL’s ‘Activities of Daily Living’ domain was more effective for Group-based exercise compared to home-based exercise.

Group-based program compared to the home-based program (at 24 weeks) presented greater improvement in CC, CST, HGS, 4 m test, gait speed, and knee strength (right knee extension 180°/s and left knee flexion 90°/s (Table 6).

## 5. Discussion

To our knowledge, this is the first study evaluating two different modes of exercise training programs; group versus home-based exercise in elderly adults with sarcopenia. The present study evaluated various parameters related to body composition, muscular, functional/physical performance and QoL. As it had been expected, our results show that exercise interventions are effective (in these parameters) for sarcopenic subjects. The one-to-one exercise model is effective in terms of patient motivation and for securing compliance; however, it is costly and requires more human resources [39,40,41]. As aging population is significantly increasing and the need for healthcare utilization is constantly rising [4], it is highly unlikely that the one-to-one (healthcare professional to patient) model will be able to serve large patient volumes. Alternative training modes, which are applicable to larger populations, are group-based and home-based exercise programs. They are considered to be more convenient and cost effective, especially for elderly populations [41,42]. However, as there is so far no evidence of whether one mode is superior to the other for sarcopenic patients, this quest formed the basis of our research question.

Τhe principal finding was that supervised group-exercise therapy was more effective than home-based exercise therapy in improving 15 out of 17 variables after 12 weeks (compared to 7 variables for the home-based group). SMMI, HGS and almost all knee strength variables (except for left knee extension 180°/s) were improved in group-based exercise. Improvement of muscle strength is important considering that sarcopenia is a disease of the muscle (muscle failure). During the 2018 Working Group (EWGSOP2) meeting, the original definition of sarcopenia was updated and low muscle strength was agreed to be the principal determinant of sarcopenia, thus, overtaking the role of low muscle mass. Indeed, muscle strength has been proven to be a better predictor of adverse outcomes compared to muscle mass [43]. Regarding the physical performance measures (TUG, 4 m and CS test), these were improved following the group-based exercise program. Improvement in physical performance and gait speed is also an important finding considering that gait speed has been shown to predict adverse outcomes related to sarcopenia [43]. These are clinically significant findings for an impaired or older population such as ours, because improvements in strength, and gait speed can have major impacts on an individual’s ability to remain independent in the community [44]. 

Home-based exercise training improved gait speed, 4 m test, TUG, CS test, SarQoL, and knee flexion strength. For this group, differences were significant compared to control group for gait speed, TUG, SMMI, HGS, knee strength and QoL. In the only published study exploring a home-based program for sarcopenic elderly community-dwellings [45], results were in agreement with this study’s findings; demonstrating benefits of home exercise in muscle strength and function (handgrip strength, single-leg standing and knee extension strength). They evaluated a 6-month home exercise program, consisting of walking and lower limb resistance exercises in sarcopenic and pre-sarcopenic elderly. Surprisingly, a 23.5% of participants in the intervention group (8 out of 34 participants) dropped out of the study, thus, causing big concerns about the study’s external validity. The strength of the present study was that no dropouts were recorded in contrast to other studies utilizing home-based programs [42,45]. A possible explanation may be due to the regular visits by the physiotherapist/instructor and the telephone counselling between visits, thus, providing ongoing support and motivation. Telephone counselling is considered effective for promoting physical activity in elderly and has the potential for being a convenient low-cost alternative compared to face-to-face contact [46]. Furthermore, all components of the program were ‘easy to perform’ and did not require any special facility or equipment.

In the long-term, at the 24 weeks follow-up, the group-based exercise program proved to have maintained most improvements compared to the home-based one. Actually 11 out of 17 measurements were improved compared to 3 measurements (for the home-based). Improvements on SMMI, TUG, gait speed, 4 m test, CS test, CC, and knee strength measurements of the group-based intervention were retained over the 6-month period; 3 months after cessation of the exercise program. In contrast, the home-based group maintained improvements, only in three functional/physical performance measures; gait speed, TUG and 4 m test. A possible explanation may be that center-based interventions usually have greater supervision and tend to motivate participants more efficiently. Support and feedback provided by the instructor may have helped participants to gauge further progress, serving as an enhanced motivator [46,47]. Furthermore, participation in group-based programs has been reported to increase adherence, improve psychological status (e.g., self-esteem), and enhance socializing [40,47,48].

As far as the control group was concerned, results did not yield any statistically significant changes among tested variables either at 12 or 24 weeks. As it was expected, health education is not enough for improving muscle strength or function in elderly with sarcopenia.

SarQoL questionnaire has been used in the current study for assessing quality of life. To our knowledge, this is the first clinical trial using the SarQoL. This questionnaire has been successfully cross-culturally validated in 6 languages, Greek included [29,49,50,51,52,53]. Evaluating the impact of sarcopenia on individuals’ QoL with a disease-specific tool is important for detecting treatment effectiveness and for observing longitudinal health changes amongst this population [54,55]. In the present study, QoL increased significantly after 12 weeks for both exercise groups compared to the control group, and remained at these improved levels after 24 weeks. In terms of the questionnaire’s domains, group-based exercise program was superior to home-based exercise only in the ADL domain. However, this difference is important since poor ADL skills are correlated with more frequent falls, poorer cognitive function, lower bone mass, frailty, disabilities and mortality [56,57]. It is today widely accepted that participation in social activities (such as a group exercise activity) contributes towards successful ageing whilst, at the same time, maintaining independence in the activities of daily living (ADLs) is important for the elderly’s self-efficacy [58]. Furthermore, the group-based program appeared to be more effective, compared to the control, yielding significant improvements in 5 out of 7 domains of the SarQoL. The home-based program showed significant difference only in one domain. Considering the characteristics of our sarcopenic sample the majority of them were sarcopenic with no severe health and/or mental conditions. It is possible that results could have been different if sarcopenic participants were older and severely sarcopenic. It would be of great interest to perform subgroup analysis of QoL between sarcopenic and severely sarcopenic older people in future studies.

As far as the study’s exercise program in concerned, this was based on simplicity, safety, feasibility, and applicability for older subjects, which are considered as the most important criteria for exercise prescription for the elderly [21]. Due to the multifactorial nature of sarcopenia, we designed a multicomponent program including progressive resistance, aerobic type, functional, and balance exercises [55,56,57,59,60]. Evidence suggests that both resistance exercise and aerobic training, leads to beneficial adaptations on muscle strength and improve physiological responses in the aging population [14,61,62]. Both programs (group-based and home-based) included the same exercises, balanced in terms of volume and intensity. This was decided in order to minimize bias on the exercise program’s content; thus, both groups received the same type and amount of exercise.

The results of the present study support the efficacy of group-based exercise in sarcopenic elderly patients, after 3 months of exercise and at 6 months follow-up. Group exercise is believed to be beneficial and its mechanisms of action are believed to be attributed to physical, psychological, and social factors [40]. The beneficial effects of group-based exercise in various populations are also supported by previous clinical studies [42,63,64]. Physical performance is suggested to improve in a group setting, probably because within group ‘comparative’ feedback improves motor learning skills [63]. In terms of the psychosocial aspect, group exercise enhances motivation, improves self-esteem and enjoyment as well as enhances socializing factors, which are all crucial for exercise adherence [48,49,65,66,67,68,69]. All these attributes are involved in the motor learning process, which forms the essence of physical performance [65]. In the current study, a high percentage (~39%) of the sarcopenic elderly lived alone and the aforementioned psychologic benefits of group participation, may have also been another reason for the more improved outcomes compared to home exercise. This could also justify the tendency towards a marginally better adherence in the group-based compared to the home-based participants (91.66% versus 87.5%).

In terms of physical performance, the home-based program yielded improvements in both, 12 and 24 weeks (although not as great as the group ones). However, for elderly who are unable or unwilling to participate in center-based group exercise, home-based exercise program could provide an effective alternative for them [70].

Additionally, effects of exercise may also depend on population characteristics (including age, gender, and lifestyle factors [71,72]. In terms of our patient profile our sample was homogenous in nearly all baseline assessment parameters (number of drugs, comorbidities, social status, education, etc.). Smoking was the only factor which reached statistical significance, and was thus different across groups. However, it does not appear to have a large impact on sarcopenia [73]. As far as the sample’s sarcopenic status is concerned, 83% were sarcopenic and the remaining participants were severely sarcopenic. We did not have any pre-sarcopenic subjects. It is not unreasonable to assume that differences in sarcopenic status may have an effect in both, exercise and assessment parameters. Unfortunately, in view of the small number of severely sarcopenic elderly in our study, we were unable to conduct a further subgroup analysis. Certainly, address this would be beneficial for future studies.

The current study provides evidence that older individuals with sarcopenia can safely and effectively participate both in structured center-based group exercise as well as in individual home-based exercise. Of major clinical importance is the result that the supervised group-based exercise is even more beneficial than the home-based program. The former was superior in all diagnostic variables for sarcopenia (SMMI, muscle strength, and physical performance) and, in addition to its cost effectiveness [42] appears to be the optimal choice. The home-based exercise could be an alternative option in case of inability to participate in such a program.

A limitation of the present study is that muscle mass was measured using BIA. Although magnetic resonance imaging (MRI), computed tomography, and dual-energy X-ray absorptiometry are the most valid and reliable clinical methods for measuring muscle mass [29,74], they have the disadvantage of being costly. BIA is a noninvasive, quick, safe and inexpensive method of measuring body composition and its use has been reported in an increasing number of studies over the last decade [9,10,11,12,13]. In addition, the European Group on Sarcopenia in Older People accepts bioelectrical impedance analysis as an option for sarcopenia assessment [43]. In this study the formula which was used (SMM (kg) = 0.566 * FFM) was validated on individual and group data and has been compared with SMM data calculated from 24 h creatinine excretion in a group of healthy subjects as well [28,75]. Another shortcoming of the study was the small number of male participants (only 7). In terms of statistical analysis, we did not perform adjustment for multiple comparisons. Clearly, longer-term interventions with larger samples are recommended to assess impacts on muscle mass, muscle strength and functional performance in elderly with sarcopenia.

## 6. Conclusions

The study’s findings provide encouraging data on the effects of exercise in sarcopenic elderly population. Sarcopenic population is capable of responding to an exercise program whether in a supervised or a home-based setting. Both approaches can be effective. However, supervised group-based exercise seems to be superior to home-based exercise therapy in almost all variables. Furthermore, the group-based program induced improvements in SMMI, TUG, gait speed, 4 m test, CS test, CC, and knee strength; which were maintained for at least 3 months after the end of the exercise program.

## Figures and Tables

**Table 1:** Exercise intervention program.

	Warm up	Resistance	Balance	Cool down
**Phase 1 (weeks 1–4)**	**Activity:** seated marching, forward walk and turn, semi-tandem walk, circle walking, sit to stand, double side arm raise, neck flexion, neck rotation, ankle—four-way **Duration:** 5 min	**Activity:** knee extensor-flexor, hip abductor-extension, ankle plantar flexors- dorsi flexors, wall push up, seated bicep curl, seated triceps extension, seated lateral shoulder raises, seated abdominal crunches **Duration:** 15 min **Reps:** 8 × 1 set **Intensity:** Easy (10–11 points in the Borg RPE)	**Activity:** walking and turning around, walking- backward- tandem-heel-toe- heel to toe-sideways, one leg stand, tandem stance **Duration:** 15 min **Reps:** 8 × 1 set **Intensity:** Easy (10–11 points in the Borg RPE)	**Activity:** forward walk/march, stretch-neck/shoulder/chest/rhomboid/, /tricep/lats/oblique, /hamstring, /gastrocnemius **Duration:** 5 min
**Phase 2 (weeks 5–8)**	**Αctivity:** As in phase 1 **Duration:** 5 min	**Activity:** As in phase 1 Added sit to stand exercise **Duration:** 20 min **Reps:** 10 × 2 sets **Intensity:** Easy (10–12 points in the Borg RPE)	**Activity:** As in phase 1 Added walking backward **Duration:** 20 min **Reps:** 10 × 2 sets **Intensity:** Easy (10–12 points in the Borg RPE)	**Activity:** As in phase 1 **Duration:** 5–10 min
**Phase 3 (weeks 9–12)**	**Activity:** As in phase 2 **Duration:** 5–10 min	**Activity:** As in phase 2 **Duration:** 20 min **Reps:** 12 × 2 sets **Intensity:** Medium to hard (12 points in the Borg RPE)	**Activity:** As in phase 2 **Duration:** 20 min **Reps:** 12 × 2 sets **Intensity:** Medium to hard (12 points in the Borg RPE)	**Activity:** As in phase 2 **Duration:** 5–10 min

Borg RPE: Borg Rating of Perceived Exertion.

**Table 2:** Baseline characteristics of sarcopenic participants (*n* = 54). SarQoL_GR: Sarcopenia Quality of Life.

Comorbidities	Group-Based	Home-Based	Control Group	Total	*p*-Value
	(*n* = 18)	(*n* = 18)	(*n* = 18)	(*n* = 54)	
mean ± SD					
Age	74.56 ± 6.04	71.17 ± 6.47	72.89 ± 8.31	72.87 ± 7.02	0.35
Body mass index (BMI) (kg/m^2^)	21.95 ± 2.18	23.24 ± 2.78	22.98 ± 2.29	22.72 ± 2.45	0.25
Drugs (*n*)	3.55 ± 1.24	3.33 ± 1.37	2.77 ± 1.66	3.22 ± 1.44	0.25
Comorbidities	2.66 ± 1.08	2.5 ± 0.92	2.83 ± 1.2	2.66 ± 1.06	0.65
Quality of life (SarQol_GR score)	57.08 ± 13.24	56.51 ± 10.64	53.21 ± 13.15	56.6 ± 12.28	0.6
Handgrip strength	17.22 ± 4.8	19.2 ± 3.6	17.43 ± 4.04	17.95 ± 4.2	0.304
Gait speed	0.87 ± 0.13	0.9 ± 0.10	0.91 ± 0.12 ±	0.89 ± 0.11	0.63
number (percentage)					
Gender					0.87
Female	16 (89%)	15 (84%)	16 (89%)	47 (87.3%)	
Male	2 (11%)	3 (16%)	2 (11%)	7 (12.7%)	
Smoking	2 (11%)	3 (16%)	8 (44%)	13 (24%)	0.04
Educational status					
Elementary	3 (16.7%)	2 (11.1%)	2 (11.1%)	7 (12.7%)	
High school	11 (61.1%)	8 (44.4%)	11 (61.1%)	30 (54.55)	0.11
University	4 (22.2%)	7 (38.9%)	5 (27.8%)	16 (29.1%)	
Marital status					
Married	11 (61.1%)	11 (61.1%)	9 (50%)	31 (56.4%)	
Widowed	6 (33.3%)	3 (16.7%)	7 (38.9%)	16 (29.1%)	0.56
Divorced	0 (0%)	2 (11.1%)	0 (0%)	2 (3.6%)	
Single	1 (5.65)	2 (11.1%)	2 (11.1%)	5 (9.1%)	
Falls	9 (50%)	7 (38.9%)	9 (50%)	25 (45.5%)	0.75

**Table 3:** Comparisons of variables among groups.

	Variables	Total Sample *	Group	Baseline	After 3 Months Interventions	6 Months Follow-Up	ANOVA (GxT)
		(*n* = 54)					*p* Value
	BMI (kg/m^2^)	22.72 ± 2.45	GE	21.95 ± 2.18	22.19 ± 2.12	22.08 ± 1.98	0.58
			HE	23.24 ± 2.78	23.44 ± 2.43	23.33 ± 2.29	
			C	22.72 ± 2.45	22.95 ± 2.08	22.64 ± 2.02	
	SMMI	5.64 ± 0.56	GE	5.7 ± 0.49	5.94 ± 0.51	5.86 ± 0.46	0.003 **
			HE	5.64 ± 0.55	5.71 ± 0.54	5.69 ± 0.5	
			C	5.58 ± 0.66	5.57 ± 0.65	5.54 ± 0.65	
	Fat free mass	25.05 ± 4.26	GE	25.59 ± 4.4	26.5 ± 4.4	25.95 ± 4.68	0.45
			HE	24.41 ± 4.9	24.73 ± 4.98	24.38 ± 3.1	
			C	25.15 ± 3.52	24.57 ± 4.66	26.45 ± 10.46	
	Calf circumference (cm)	32.76 ± 2.23	GE	33.17 ± 2.47	33.61 ± 2.32	33.72 ± 2.34	<0.001 **
			HE	32.67 ± 2.52	32.76 ± 2.58	32.7 ± 2.51	
			C	32.44 ± 1.65	32.38 ± 1.71	32.27 ± 1.84	
	TUG (sec)	10.06 ± 1.98	GE	10.04 ± 2.68	8.27 ± 2.07	8.61 ± 2.48	<0.001 **
			HE	9.67 ± 1.29	8.47 ± 1.22	8.82 ± 1.37	
			C	10.48 ± 1.75	10.61 ± 1.67	10.52 ± 1.8	
	4 m test (s)	4.52 ± 0.65	GE	4.66 ± 0.84	3.34 ± 0.49	3.17 ± 0.75	<0.001 **
			HE	4.47 ± 0.53	3.75 ± 0.54	3.71 ± 0.70	
			C	4.42 ± 0.53	4.42 ± 0.69	4.64 ± 0.76	
	Gait speed (m/s^2^)	0.89 ± 0.11	GE	0.87 ± 0.13	1.21 ± 0.15	1.32 ± 0.3	0.45
			HE	0.9 ± 0.10	1.08 ± 0.15	1.11 ± 0.2	
			C	0.91 ± 0.12	0.92 ± 0.15	0.87 ± 0.15	
	Chair stand test (s)	13.59 ± 3.52	GE	13.72 ± 5.07	10.78 ± 3.94	11.15 ± 4.82	<0.001 **
			HE	12.29 ± 1.54	10.9 ± 1.98	11.41 ± 2.17	
			C	14.76 ± 2.72	14.38 ± 3.19	14.61 ± 3.22	
	Handgrip strength (kg)	17.95 ± 4.2	GE	17.22 ± 4.8	20.58 ± 4.29	20.07 ± 4.35	<0.001 **
			HE	19.2 ± 3.6	19.33 ± 4.51	18.68 ± 3.34	
			C	17.43 ± 4.04	17.92 ± 4.14	17.34 ± 3.77	
**Isokinetic measurements**	Right knee ext 90° (Nm/kg)	36.46 ± 9.1	GE	45.21 ± 12.87	51.69 ± 12.87	50.09 ± 11.94	0.003 *
			HE	47.73 ± 9.75	51.61 ± 12.66	50.48 ± 11.49	
			C	44.48 ± 12.38	43.15 ± 10.76	41.88 ± 10.23	
	Right knee	36.46 ± 9.1	GE	34.61 ± 10.06	39.87 ± 12.03	39.03 ± 10.51	<0.001 **
	ext 180° (Nm/kg)		HE	38.31 ± 7.89	39.94 ± 11.81	37.69 ± 9.18	
			C	36.47 ± 9.35	33.63 ± 8.97	33.35 ± 7.7	
	Right knee	30.74 ± 10.1	GE	30.15 ± 10.76	38.57 ± 7.52	38.25 ± 7.58	0.002 *
	flex 90° (Nm/kg)		HE	31.67 ± 8.62	34.59 ± 10.12	35.05 ± 8.87	
			C	30.4 ± 11.48	27.15 ± 10.43	26.75 ± 10.36	
	Right knee	26.92 ± 7.96	GE	27.38 ± 6.84	34.18 ± 7.1	32.62 ± 6.72	<0.001 **
	flex 180° (Nm/kg)		HE	27.99 ± 7.6	29.6 ± 7.94	29.08 ± 7.29	
			C	25.38 ± 9.43	23.69 ± 9	25.53 ± 9.48	
	Left knee ext 90° (Nm/kg)	43.06 ± 10.66	GE	45.59 ± 10.9	50.4 ± 10.91	49.66 ± 10.03	0.03 *
			HE	44.51 ± 7.59	47.6 ± 10.1	47.24 ± 10.6	
			C	39.07 ± 12.34	39.67 ± 11.1	36.43 ± 10.03	
	Left knee ext 180° (Nm/kg)	34.4 ± 8.05	GE	34.86 ± 8.75	38.71 ± 8.88	39.03 ± 10.51	0.17
			HE	36.29 ± 6.79	38.93 ± 5.71	37.69 ± 9.18	
			C	32.05 ± 8.33	31.17 ± 8.56	33.35 ± 7.7	
	Left knee flex 90° (Nm/kg)	29.54 ± 9.25	GE	28.94 ± 9.74	36.26 ± 10.26	34.5 ± 8.6	<0.001 **
			HE	31.67 ± 8.62	33.51 ± 7.81	32.4 ± 7.82	
			C	28.06 ± 9.51	29.09 ± 9.47	26.14 ± 10.44	
	Left knee flex 180° (Nm/kg)	23.58 ± 7.78	GE	26.95 ± 7.69	31.39 ± 8.03	29.17 ± 7.47	0.055
			HE	26.86 ± 7.5	31.56 ± 7.23	28.71 ± 7.29	
			C	23.58 ± 8.11	24.41 ± 8.78	22.29 ± 9.07	
	Quality of life (SarQoL)	56.6 ± 12.28	GE	57.08 ± 13.24	64.37 ± 11.77	61.4 ± 15.73	<0.001 **
			HE	56.51 ± 10.64	59.93 ± 11.28	58.5 ± 11.23	
			C	53.21 ± 13.15	58.44 ± 12.74	49.22 ± 10.49	

SMMI: Skeletal muscle mass index; BMI: Body mass index; SarQoL: Sarcopenia quality of life questionnaire; TUG: Timed up and Go test; Data expressed as mean (SD); GE: group-based exercise; HE: home-based exercise; C: control group; GxT: group x time; * statistically significant (*p* < 0.05), ** highly statistically significant (*p* < 0.001).

**Table 4:** Mean difference scores for each group across time.

	Variable	Difference Means	Exercise-Based Group	Home-Based Group	Control Group
	SMMI	baseline-12wks	−0.24 *	−0.07	0.01
		baseline-24wks	−0.16 *	−0.05	0.03
	BMI	baseline-12wks	−0.24	−0.19	0.02
		baseline-24wks	−0.13	−0.08	0.34
	TUG	baseline-12wks	1.77 *	1.20 *	−0.13
		baseline-24wks	1.42 *	0.84 *	−0.14
	Handgrip strength (kg)	baseline-12wks	−3.36 *	−0.13	−0.13
		baseline-24wks	−2.85 *	0.52	0.52
	Gait speed	baseline-12wks	−0.33 *	−0.17 *	−0.01 *
		baseline-24wks	−0.44 *	−0.21 *	0.03 *
	4 m test	baseline-12wks	1.32 *	0.71 *	0
		baseline-24wks	1.49 *	0.75 *	−0.22
	Chair stand test	baseline-12wks	2.94 *	1.39 *	0.37
		baseline-24wks	2.57 *	1.87	0.14
	Quality of life (SarQoL)	baseline-12wks	−7.28 *	−3.41 *	2.19
		baseline-24wks	−4.32	−1.99	3.99
	Calf circumference (cm)	baseline-12wks	−0.44 *	−0.11	0.05
		baseline-24wks	−0.55 *	−0.05	0.16
**Isokinetic measurements**	Right knee ext 90°/s	baseline-12wks	−6.47 *	−3.88	1.32
		baseline-24wks	−4.87 *	−2.75	2.59
	Right knee ext 180°/s	baseline-12wks	−5.26 *	−1.62	2.83
		baseline-24wks	−4.42 *	0.62	3.11
	Right knee flex 90°/s	baseline-12wks	−8.42 *	−2.91	3.25
		baseline-24wks	−8.09 *	−3.37	3.64
	Right knee flex 180°/s	baseline-12wks	−6.8 *	−4.69 *	−0.82
		baseline-24wks	−1.61	−1.84	1.28
	Left knee ext 90°/s	baseline-12wks	−4.80 *	−3.08	−0.60
		baseline-24wks	−4.06	−2.73	2.63
	Left knee ext 180°/s	baseline-12wks	−3.85	−2.64	0.87
		baseline-24wks	−4.17	−1.40	−1.29
	Left knee flex 90°/s	baseline-12wks	−7.31 *	−1.88	−1.03
		baseline-24wks	−5.55 *	−0.77	1.92
	Left knee flex 180°/s	baseline-12wks	−4.44 *	−4.69 *	−0.83
		baseline-24wks	−2.22	−1.84	1.28

SMMI: Skeletal muscle mass index; BMI: Body mass index; SarQoL: Sarcopenia quality of life questionnaire; TUG: Timed up and Go test; HGS: Handgrip strength. * statistically significant differences

**Table 5:** Between-group comparisons at 12 weeks.

Variable	ANOVA (*p* Value)	Post Hoc (Bonferroni) Analysis		
		Group-Based vs. Home-Based Exercise Group	Group-Based vs. Control	Home-Based vs. Control
**Skeletal muscle mass index**	≤0.001	≤0.001	≤0.001	NS
**Calf circumference**	≤0.001	≤0.001	≤0.001	NS
**Chair stand test**	≤0.001	≤0.05	≤0.001	NS
**Handgrip strength**	≤0.001	≤0.001	≤0.05	NS
**Right knee extension 90°/s**	0.005	NS	≤0.05	NS
**Right knee extension 180°/s**	≤0.001	NS	≤0.001	NS
**Right knee flexion 90°/s**	0.004	NS	≤0.05	NS
**Right knee flexion 180°/s**	≤0.001	≤0.001	≤0.001	NS
**Left knee extension 180°/s**	0.01	NS	0.02	NS
**Left knee flexion 90°/s**	0.004	0.02	≤0.05	ΝS
**4m test**	≤0.001	0.003	≤0.001	≤0.001
**TUG**	≤0.001	NS	≤0.001	≤0.001
**Gait speed**	≤0.001	≤0.001	≤0.001	≤0.001
**Quality of life (SarQoL)**	≤0.001	NS	≤0.001	≤0.05
**Physical and mental health (Domain 1)**	≤0.001	NS	≤0.05	NS
**Locomotion (Domain 2)**	0.014	NS	≤0.05	NS
**Functionality (Domain 4** **)**	0.03	NS	≤0.05	NS
**Activities of daily living (Domain 5)**	≤0.001	≤0.05	≤0.001	≤0.05
**Fears (Domain 7)**	0.07	NS	≤0.05	NS

TUG: Timed up and Go test; NS: non-significant differences; body mass index, body composition (SarQoL-Domain 3) and leisure activities (SarQoL-Domain 6) did not yield significant differences.

**Table 6:** Between-group comparisons at 24 weeks.

Variable	ANOVA (*p* Value)	Post Hoc (Bonferroni) Analysis		
		Group-Based vs. Home-Based Exercise Group	Group-Based vs. Control	Home-Based vs. Control
**Skeletal muscle mass index**	≤0.001	NS	≤0.001	NS
**Calf circumference**	≤0.001	≤0.05	≤0.001	NS
**Chair stand test**	≤0.001	≤0.001	≤0.05	NS
**Handgrip strength**	≤0.001	0.001	≤0.05	NS
**Right knee extension 90°/s**	0.005	NS	≤0.05	NS
**Right Knee extension 180°/s**	0.005	≤0.05	≤0.01	NS
**Right knee flexion 90°/s**	0.003	NS	≤0.05	NS
**Right knee flexion 180°/s**	0.013	NS	≤0.05	NS
**Left knee extension 180°/s**	0.49	NS	≤0.001	NS
**Left knee flexion 90°/s**	≤0.001	≤0.05	≤0.001	ΝS
**4 m test**	<0.001	0.003	<0.001	<0.001
**TUG**	≤0.001	NS	≤0.001	≤0.05
**Gait speed**	≤0.001	≤0.05	≤0.001	≤0.001
**Quality of life (SarQoL)**	0.002	NS	≤0.05	≤0.05
**Physical and mental health (Domain 1)**	0.05	NS	≤0.05	NS
**Locomotion (Domain 2)**	0.01	NS	≤0.05	NS
**Activities of daily living (Domain 3)**	0.02	NS	≤0.05	NS

TUG: Timed up and Go test; NS: non-significant differences; body mass index, Body Composition (SarQoL-Domain 3), Functionality (SarQoL-Domain 4), Leisure activities (SarQoL-Domain 6) and Fears (SarQoL-Domain 7) did not yield significant differences.
